# As-Casting Structure and Homogenization Behavior of Ta-Containing GH4151 Ni-Based Superalloy

**DOI:** 10.3390/ma18081742

**Published:** 2025-04-10

**Authors:** Tianliang Cui, Xingfei Xie, Wugang Yu, Jinglong Qu, Shaomin Lyu, Jinhui Du

**Affiliations:** 1 China Iron and Steel Research Institute Group Co., Ltd., Beijing 100081, China; 15030108050@139.com (T.C.); lsmleon@163.com (S.L.); superalloy_1@163.com (J.D.); 2 Beijing GAONA Materials & Technology Co., Ltd., Beijing 100081, China; 3 Sichuan Gaona Forging Co., Ltd., Deyang 618000, China; 4 NCS Testing Technology Co., Ltd., Beijing 100081, China; ellt@163.com

**Keywords:** Ni-based superalloy, microstructure and microsegregation, diffusion coefficient, activation energy, hot deformation

## Abstract

In this paper, the as-cast microstructure, microsegregation, the kinetics of secondary precipitation phase, and thermal deformation behavior in Ta-containing GH4151 alloy (Ta-GH4151) were studied using optical microscope (OM), scanning electron microscope (SEM), electron probe (EPMA), differential scanning calorimetry (DSC), mechanical testing and simulation (MTS) and electron backscattering diffraction (EBSD). The results indicate that Ti, Ta, Nb and Mo are mainly distributed in the interdendritic region and exhibit negative segregation characteristics, while Cr and W are mainly distributed in the dendritic arm region and exhibit positive segregation characteristics. The initial dissolution temperatures for Laves phase, eutectic (γ + γ′) and η phase are 1140–1150 °C, 1150–1160 °C and 1170–1180 °C, respectively. The diffusion activation energies of Nb, Ta and W are 313 kJ/mol, 323 kJ/mol and 345 kJ/mol, respectively. The hot deformation activation energy of Ta-GH4151 alloy after homogenization is 1694.173 kJ/mol. Based on the constitutive equation and hot processing map, the optimum hot deformation temperature and strain rate range are determined to be 1160–1170 °C/0.3–1 s^−1^. The addition of Ta not only increases the redissolution temperature of the Laves phase, eutectic (γ + γ′) and η phase but also increases the segregation of Nb, Ta and W, diffusion activation energy and homogenization. The results are expected to provide a more comprehensive understanding of the modification and accelerated application of GH4151 alloy.

## 1. Introduction

Cast and wrought (C&W) superalloys have been widely used in advanced aero-engines due to their excellent high-temperature strength, durability, simple preparation process and low cost [1,2,3]. With the development of aviation technology, higher requirements are imposed on the heat-resistant temperature of superalloys used in turbine disks. However, Europe and the United States have successfully developed turbine disk materials that can resistant 815 °C [4]. In order to reduce the manufacturing cost of high-performance aero-engine, there is an urgent need to develop cast and wrought superalloys that can resistant temperatures of more than 800 °C.

Supplementary alloying is an effective way to improve the high-temperature properties of superalloys [5]. It has been reported that the addition of Ta can increase the γ/γ′ lattice mismatch of superalloys, improve the stability of the γ′ phase structure, and significantly improve the high-temperature properties [6,7]. GH4151 alloy is a highly alloying and hard-to-deform Ni-base superalloy [1,8,9]. The age-precipitation strengthening elements Al, Ti, and Nb exceed 10 wt.%. The mass fraction of the γ′ phase at equilibrium is up to 55%. Its service temperature can reach 750–800 °C, which is one of the most hard-to-deform superalloys in terms of service temperature [10,11]. Adding alloying (adding the Ta element) on this basis will be expected to break through the service temperature of 800 °C. Li et al. [12] showed that the maximum homogenization temperature of as-cast GH4151 alloy was as high as 1180–1200 °C, which was close to the initial melting temperature of the alloy due to the serious segregation of as-cast GH4151 alloy. The addition of Ta will further increase the segregation, so it is challenging to study the homogenization of Ta-containing GH4151 alloy (Ta-GH4151).

Recently, the homogenization of Ta-containing superalloys has been reported. Reed et al. [13,14] found that the addition of Ta can reduce the formation trend of freckles and hot cracks during the directional solidification of IN792 alloy. Kearsey et al. [15] showed that in single-crystal superalloys, Ta tends to distribute to the interdendritic arm region, increasing the content of segregated phases. Song et al. [16] showed that Ta is the main forming element of the γ′ phase. The primary dendrite arm spacing (PDAS) and the volume fraction of the eutectic (γ + γ′) of nickel-based single-crystal superalloys increase significantly with the increase of the Ta/Al ratio. Peng et al. [17] suggested that Ta increased the solidification range of superalloys, resulting in the volume fraction of MC carbides and the eutectic (γ + γ′). In contrast, Gao et al. [18] suggested that the addition of Ta would not affect as-cast organization characteristics such as grain size, secondary dendrite arm spacing, and carbide distribution. On the other hand, with the increase of the total content of precipitation-strengthening elements in superalloys, the difficulty of hot deformation increases. For example, Lypchanskyi et al. studied the Waspaloy alloy with a total precipitation strengthening element of 4.5% and measured its thermal activation energy of 630,170,000 J/mol [19]; the thermal activation energy of GH4151 alloy with 9.6% precipitation strengthening elements reported by Jia et al. is 1,086,580 J/mol [20]. It can be inferred that further increasing the strengthening precipitation element Ta on the basis of GH4151 alloy will inevitably increase the difficulty of hot deformation. However, there is a lack of reports on the effect of Ta on the homogenization and thermal processing of highly alloyed and hard-to-deform superalloys.

In this paper, the element segregation and microstructure evolution of Ta-containing GH4151 alloy during homogenization are studied. The redissolution kinetics equations of the Laves phase, eutectic (γ + γ′), and η phase are established. The diffusion coefficient and activation energy of Nb, Ta, and W elements are calculated by the residual segregation index. The hot deformation behavior and machinability of homogenized Ta-GH4151 alloy are studied by hot compression. The results will provide a more comprehensive understanding of the modification and accelerated application of the GH4151 alloy.

## 2. Experimental

The ∅150 mm Ta-GH4151 ingot was prepared by vacuum induction melting (VIM). The chemical constituents (nominal composition) are shown in Table 1 (Beijing GAONA Materials & Technology Co., Ltd., Beijing 100081, China). To ensure the initial microstructure’s consistency and homogeneity, experimental samples were taken from the 1/3R position of the ingot’s head cross-section. For each experiment, two samples were tested. If results exhibited large deviations, 1–3 more samples were tested until the results stabilized. The size of the optical microstructure sample is ∅8 × 6 mm ± 0.1 mm. Before the test, the sample was polished using 120#, 300#, 800#, 1500#, and 200# sandpaper. DSC test sample size was ∅5 × 0.3 mm ± 0.1 mm. Before the DSC experiment, the heating curve of the empty crucible was used as the baseline. The DSC experimental curve was obtained by subtracting the baseline from the heating curve of the sample and the crucible (NETZSCH STA 449 F3 Jupiter, Bavaria, Germany). The temperature range of the DSC test was 1000–1400 °C, and the heating rate was 10 °C/min. The heat treatment test was carried out in a muffle furnace with a temperature control of ±5 °C, sample size ∅8 × 10 mm ± 0.1 mm. The samples were held at 1140 °C, 1150 °C, 1160 °C, 1170 °C, 1180 °C, 1190 °C, and 1200 °C for 30 min and immediately quenched in water to maintain the high-temperature microstructure. To investigate the effect of homogenization parameters on the dissolution of the segregation secondary phase, the samples were held at 1140 °C and 1160 °C for 2 h, 4 h, 8 h and 12 h, and held at 1180 °C and 1195 °C for 4 h, 6 h, 8 h and 16 h. To investigate the effect of homogenization parameters on the dissolution of the diffusion of Nb, Ta, and W elements, the samples were held at 1180 °C and 1195 °C for 4 h, 6 h, 8 h and 12 h, and held at 1215 °C for 2 h, 4 h and 8 h. After homogenization, the isothermal unidirectional hot compression experiment was carried out by MTS. The size of the MTS sample was ∅8 × 12 mm ± 0.1 mm. The hot compression process parameters are shown in Table 2, and water quenching was performed immediately after the experiment to retain the high-temperature microstructure.

The optical microscopic samples were etched with 30 g CuCl_2_ + 200 mL Hcl + 300 mL C_2_H_6_O mixed solution for 60 s. SEM samples were using electrolytic polishing and electrolytic corrosion. The electrolytic polishing system was H_2_SO_4_ + 80% CH_3_OH solution at 20 V for 20 s. The electrolytic corrosion system was 30 mL H_3_PO_4_ + 3 g CrO_3_ + 2 mL H_2_SO_4_ corrosive solution at 5 V for 2 s. The transmission electron microscope (TEM) samples were electropolished with −10% perchloric acid ethanol solution at −25 °C/80 mA. The optical microstructure was observed by the Olympus GX71 microscope(Olympus Corporation, Tokyo, Japan). All etchants are from Sinopharm Chemical Reagent Co., Ltd., Shanghai, China. The EPMA samples were prepared by mechanical polishing + ultrasonic cleaning, and the samples were baked at high temperatures for 10 min prior to the experiments. The microstructure was analyzed by a JSM-7800F field emission SEM equipped with an EDS (JEOL Ltd., Tokyo, Japan). The orientation characteristics of grain structure were analyzed by a JSM-7200F field emission scanning electron microscope equipped with an EBSD detector (JEOL Ltd., Tokyo, Japan). The EBSD organization was analyzed using Channel 5 (v5.12). The information on alloy element distribution was quantitatively analyzed by JEOL model JXA-8530F electron probe microanalyzer (EPMA, JEOL Ltd., Tokyo, Japan)). The microstructure of Ta-GH4151 alloy was analyzed using a G^2^ F20 TEM at 200 kV (Thermo Fisher Scien-tific Inc., Massachusetts, USA), with bright field imaging (BF) and selected—area electron diffraction (SAED). 

## 3. Results and Discussion

### 3.1. As-Cast Microstructure and Element Segregation

**Figure 1:**
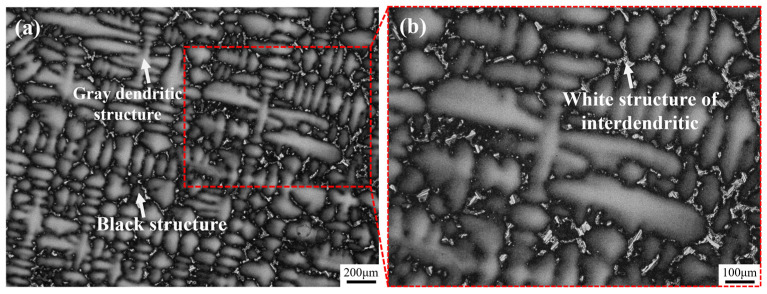
OM images of as-cast Ta-GH4151 alloy at 50× (**a**) and 100× (**b**) magnification.

Figure 1 shows the as-cast microstructure of Ta-GH4151 alloy. In this microstructure, the gray dendritic structure is the dendritic arm region, the black structure between the dendrites arm is the interdendritic region, and the white structure in the interdendritic region is the secondary phase structure of solidification segregation. The average value of secondary dendrite arm spacing (SDAS) was 61.34 μm as determined by quantitative analysis of SDAS using Image J (v1.8.0.345) software. Li et al. [12] showed that the average SDAS of ∅120 mm as-cast GH4151 alloy was 35.48 μm, while the average SDAS of ∅240 mm as-cast GH4151 alloy was 73.66 ± 5.3 μm. The increase in SDAS is related to the aggravation of element segregation in the alloy [21]. However, SDAS is also significantly affected by the cooling rate. Therefore, it may not be accurate enough to evaluate the degree of segregation only by the SDAS value. To comprehensively understand the segregation of the alloy, it is necessary to analyze the composition of the dendritic structure.

**Figure 2:**
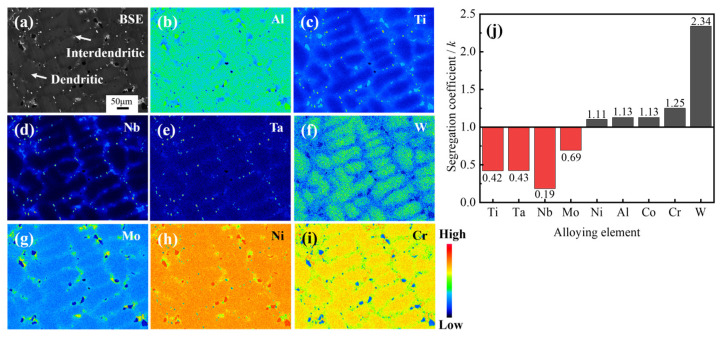
Dendritic structure by EPMA (**a**–**i**) and equilibrium partition coefficient k (**j**) of as-cast Ta-GH4151 alloy.

During the solidification process, the redistribution of solute elements at the solid-liquid interface leads to differences in the composition of the dendritic arm and interdendritic regions [22,23]. Figure 2 illustrates the elemental distribution of the as-cast structure of the Ta-GH4151 alloy, from which the segregation coefficients (*k*) for each element were calculated. The *k* is defined as the ratio of the average element content in the dendrite arm region to that in the interdendritic region [24]. As illustrated in Figure 2a–i, the distribution of Ni, Al, and Cr elements in the alloy is relatively uniform. The segregation coefficient values of *k*_Nb_, *k*_Al_ and *k*_Cr_ are close to 1, indicating that there is no significant difference in the distribution of these elements in the dendrite arm and interdendritic regions. Conversely, Ti, Ta, Nb, and Mo elements show negative segregation characteristics (*k* < 1), mainly enriched in the interdendritic region. The Nb element’s segregation is significantly higher than that of Ti, Ta, and Mo. The segregation coefficient of Nb in Ta-GH4151 alloy is *k*_Nb_ = 0.19, which is much lower than that in GH4151 alloy (*k*_Nb_ = 0.4) [10]. The elements Cr and W show positive segregation (*k* > 1) and are mainly enriched in the dendritic arm region, where the segregation tendency of W is particularly significant. The segregation coefficient of W in Ta-GH4151 alloy is *k*_W_ = 2.34, which is much lower than that in GH4151 alloy (*k*_W_ = 1.94) [10]. These findings indicate that the addition of Ta significantly enhances the segregation of elements in Ta-GH4151 alloy, which may have an important influence on the microstructure and macroscopic properties of the alloy.

**Figure 3:**
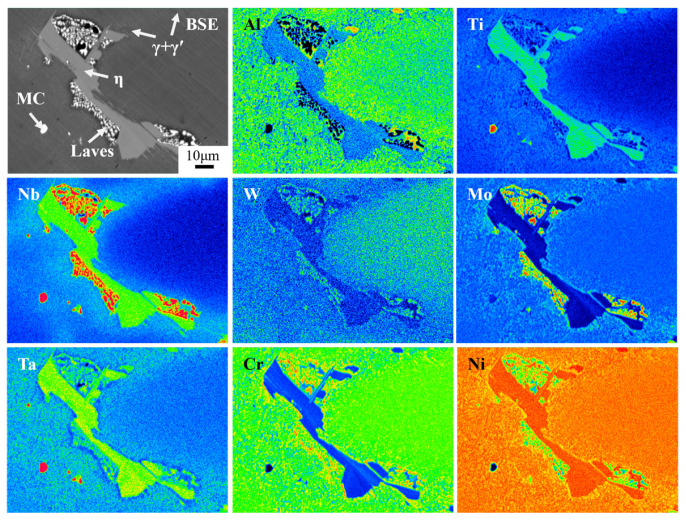
Secondary precipitates phase of as-cast Ta-GH4151 alloy by EPMA.

**Figure 4:**
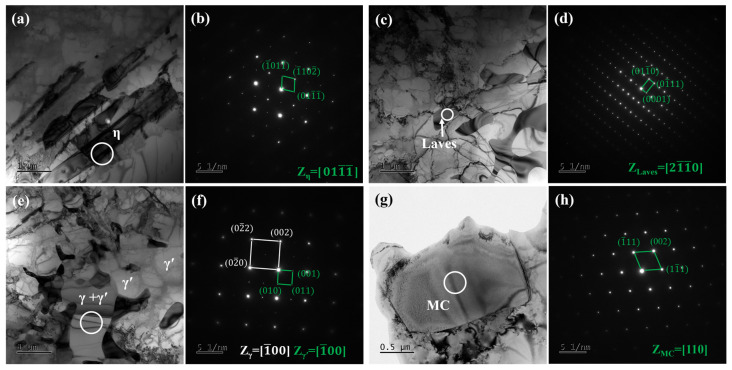
Microstructure of as-cast Ta-GH4151 alloy by TEM: (**a**) BF of η phase; (**b**) SAED corresponding to the white circular region in (**a**); (**c**) BF of Laves phase; (**d**) SAED corresponding to the white circular region in (**c**); (**e**) BF of eutectic (γ + γ′); (**f**) SAED corresponding to the white circular region in (**e**); (**g**) BF of MC; (**h**) SAED corresponding to the white circular region in (**g**).

Figure 3 shows the SEM microstructure of secondary precipitates in as-cast Ta-GH4151 alloy. Figure 4 shows the TEM microstructure of secondary precipitates in as-cast Ta-GH4151 alloy. The type of precipitates in as-cast Ta-GH4151 alloy was determined by SEM micrographs, SEM-EDS composition analysis, TEM micrographs, and TEM-SAED in combination. It can be seen that the as-cast GH4151 alloy contains four secondary precipitates. Table 3 shows the compositional characteristics of the four precipitated phases. The sunflower-like structure rich in Al, Ti, Nb and Ta is eutectic (γ + γ′) [25,26]. The plate-like structure rich in Ti, Nb and Ta is η phase, and the element ratio Ni/(Ti, Nb, Ta) ≈ 2.9 [27]. The continuous small block structure rich in Cr, Co, Mo, W, Nb and Ta is the Laves phase [8]. The block structure rich in Nb, Ta and Ti is the MC phase, namely (Nb, Ta, Ti) C phase [26,28]. The area fractions of η phase, eutectic (γ + γ′) and Laves phase in as-cast Ta-GH4151 alloy were 3.05%, 3.62% and 2.89%, respectively.

According to previous reports the solidification sequence of as-cast GH4151 alloy is: L (Liquid phase)→L+ γ (1330 °C), L→L+ MC (1290 °C), L→L+ Laves (1190 °C), L→L+ η (1170 °C), L→L+ (γ + γ′) (1150 °C) [1]. The experimental results indicate that Ta is a negative segregation element and is the forming element of the Laves phase, eutectic (γ + γ′), η phase and MC phase. During the solidification of the Ta-GH4151 alloy, the Ta element is squeezed into the interdendritic region and replaces the positions of Ti and Nb atoms, forming a secondary segregation phase rich in Ta, which leads to the increase of element segregation.

### 3.2. Effect of Homogenization on Microstructure Evolution

**Figure 5:**
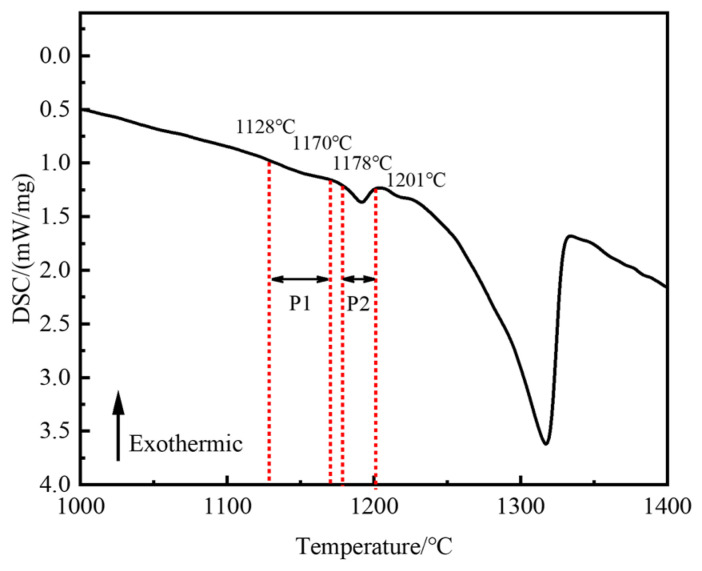
DSC curve of as-cast Ta-GH4151 alloy during the heating process.

Homogenization is an effective method of eliminating elemental segregation and secondary precipitation phases in alloys and improving hot workability [29]. In order to prevent the rapid dissolution of secondary precipitated phases at high temperatures from producing caverns, it is crucial to determine their initial dissolution temperature. Figure 5 presents the DSC analysis results of the as-cast Ta-GH4151 alloy. Two obvious endothermic peaks were observed during the heating process. The temperature range of the P1 peak is wide. The initial redissolution temperature is 1128 °C, and the final re-dissolution temperature is 1178 °C. Therefore, it can be considered that the P1 peak is the superimposed endothermic peak of the eutectic (γ + γ′) and the Laves phase. The P2 peak has an initial dissolution temperature of 1178 °C and a final dissolution temperature of 1201 °C. It is inferred that the P2 peak corresponds to the re-dissolution endothermic peak of the η phase.

**Figure 6:**
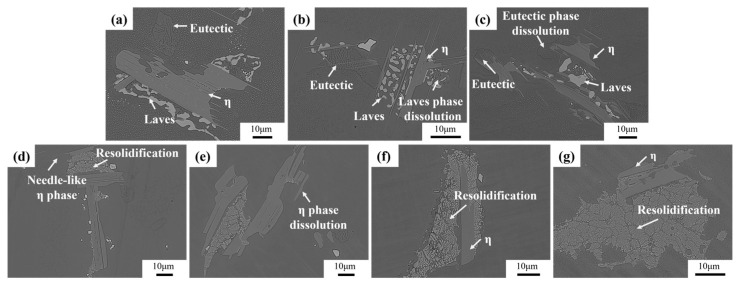
Microstructures of the Ta-GH4151 alloy at (**a**) 1140 °C, (**b**) 1150 °C, (**c**) 1160 °C, (**d**) 1170 °C, (**e**) 1180 °C, (**f**) 1190 °C, (**g**) 1200 °C for 30 min and then quenched in water.

Figure 6 illustrates the microstructural changes of as-cast Ta-GH4151 alloy in isothermal water quenching experiments at temperatures ranging from 1140 °C to 1200 °C. At 1140 °C, the morphology of the secondary precipitated phase remains relatively stable and does not change significantly. When the temperature increases to 1150 °C, the Laves phase dissolves slightly. When the temperature increases to 1160 °C, the sharp-angle characteristics of the eutectic (γ + γ′) disappear. It is shown that the eutectic (γ + γ′) is significantly dissolved at 1160 °C. When the temperature is raised to 1180 °C, the needle-like η phase completely disappears, indicating that the η phase dissolves at 1180 °C. The results of these isothermal quenching experiments are highly consistent with the experimental phenomena of DSC. Therefore, it can be determined that the initial dissolution temperature of the Laves phase is 1140–1150 °C, the initial dissolution temperature of the eutectic (γ + γ′) is 1150–1160 °C, and the initial dissolution temperature of the η phase is 1170–1180 °C.

**Figure 7:**
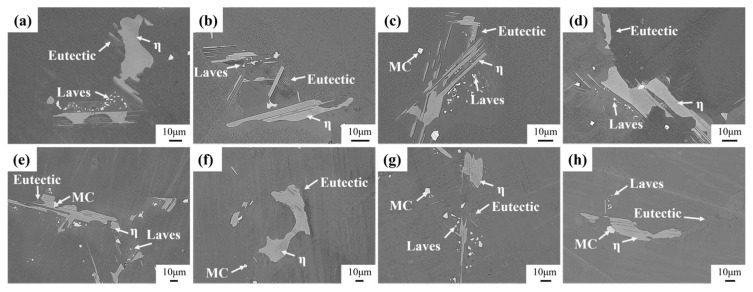
Microstructure evolution of the precipitated phase of (**a**) 1140 °C for 2 h, (**b**) 1140 °C for 4 h, (**c**) 1140 °C for 8 h, (**d**) 1140 °C for 12 h, (**e**) 1160 °C for 2 h, (**f**) 1160 °C for 4 h, (**g**) 1160 °C for 8 h, (**h**) 1160 °C for 12 h homogenized.

High-alloyed superalloys typically undergo a staged homogenization strategy to target the elimination of secondary precipitated phases [30]. Figure 7 shows the evolution of secondary precipitation phases of homogenized Ta-GH4151 alloy at 1140 °C and 1160 °C. During homogenization at 1140 °C, the Laves phase gradually dissolves with increasing holding time. The eutectic (γ + γ′) slowly dissolves at 1160 °C. The η phase does not exhibit significant dissolution behavior under both homogenization treatment conditions at 1140 °C and 1160 °C.

**Figure 8:**
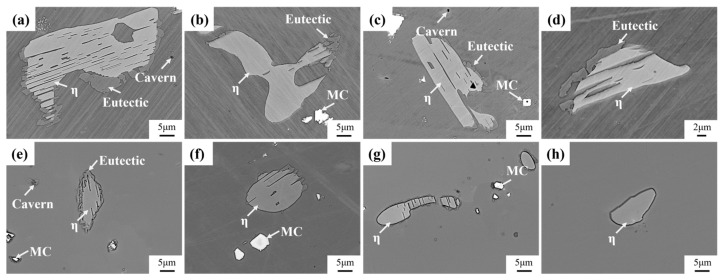
Microstructure evolution of the precipitated phase of (**a**) 1180 °C for 4 h, (**b**) 1180 °C for 6 h, (**c**) 1180 °C for 8 h, (**d**) 1180 °C for 16 h, (**e**) 1195 °C for 4 h, (**f**) 1195 °C for 6 h, (**g**) 1195 °C for 8 h, (**h**) 1195 °C for 16 h homogenized.

Figure 8 shows the evolution of the secondary precipitation phase of Ta-GH4151 alloy after homogenization at 1180 °C and 1195 °C. At 1180 °C, the η phase begins to dissolve gradually, but the dissolution rate is relatively slow. The Laves phase is almost completely dissolved, while the eutectic (γ + γ′) shows a rapid dissolution behavior. When the homogenization temperature is increased to 1195 °C, the η phase, eutectic (γ + γ′), and Laves phase all show rapid dissolution characteristics. It is noteworthy that the formation of caverns was observed during homogenization at 1180 °C and 1195 °C, which could be caused by the rapid dissolution back of the Laves phase. This phenomenon underscores the importance of when formulating the homogenization treatment scheme, the homogenization temperature of the first stage should be controlled below 1180 °C to avoid microstructure defects caused by the rapid dissolution of the secondary precipitation phase.

To predict the homogenization dissolution process of Laves phase, eutectic (γ + γ′), and η phase, a secondary phase dissolution kinetic model based on Johnson-Mehl-Avrami-Kolmogorov (JMAK), as shown in Equation (1): [9,31,32,33]:

(1) $$X=1-\exp\left(-Kt^{n}\right)=\left(S_{0}-S\right)/S_{0}$$

where *X* is the dissolution fraction of the precipitated phase, *n* is the Avrami index, *K* is the temperature constant, *t* is the time (h), *S*_0_ is the fraction of the initial precipitated phase, and *S* is the residual fraction. Taking the logarithm of both sides of Equation (1) yields Equation (2) [9,31,32,33]:

(2) $$ln\left[ln\left(\frac{1}{1-X}\right)\right]=lnK+nlnt$$

where the values of ln*K* and ln*t* can be obtained by the slope and intercept of $ln\left[ln\left(\frac{1}{1-X}\right)\right]$ vs. ln*t* [9,31,32,33].

**Figure 9:**
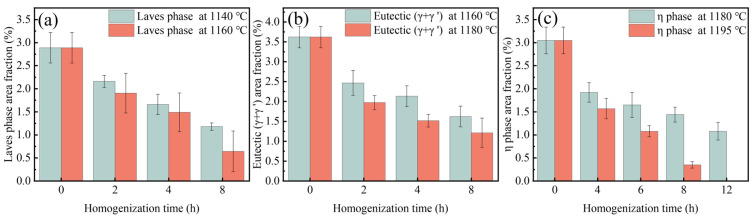
Area fraction of residual (**a**) Laves phase, (**b**) eutectic (γ + γ′) and (**c**) η phase with the change in holding time during specific steps.

**Figure 10:**
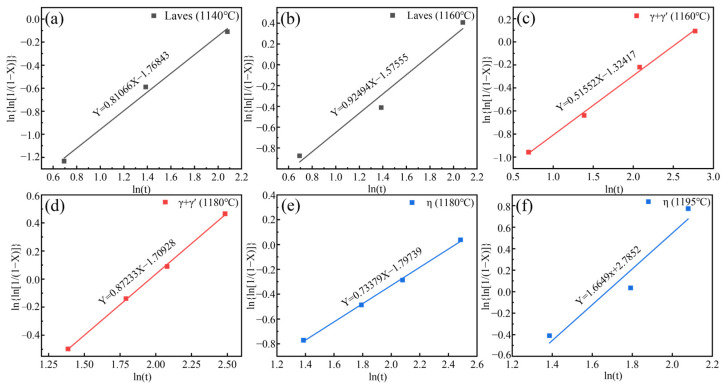
Avrami plots for dissolution of precipitated phase at different temperatures (**a**,**b**) Laves phase, (**c**,**d**) eutectic (γ + γ′), (**e**,**f**) η phase.

Figure 9 shows the area fraction of residual Laves phase, eutectic (γ + γ′) and η phase in Ta-GH4151 alloy at different holding times. Figure 10 shows the relationship of $ln\left[ln\left(\frac{1}{1-X}\right)\right]$ vs. ln*t*. The relationship between the area fraction of the residual precipitated phase and the holding time is brought into formula (2) to obtain the Avrami diagram of the dissolution of the secondary precipitated phase at different temperatures and the kinetic equation of the dissolution of the precipitated phase is calculated, such as Equations (3)–(8):

(3) $$1140\;{}^{\circ}C:\;X_{Laves}=1-exp(-1.768t^{0.811})$$

(4) $$1160\;{}^{\circ}C:\;X_{Laves}=1-exp(-1.576t^{0.925})$$

(5) $$1160\;{}^{\circ}C:\;X_{\gamma +\gamma \prime }=1-exp(-1.324t^{0.516})$$

(6) $$1180\;{}^{\circ}C:\;X_{\gamma +\gamma \prime }=1-exp(-1.709t^{0.872})$$

(7) $$1180\;{}^{\circ}C:\;X_{\eta }=1-exp(-1.658t^{0.734})$$

(8) $$1195\;{}^{\circ}C:\;X_{\eta }=1-exp(-0.062t^{1.665})$$

Assuming the *X* = 99% precipitated phase is completely eliminated, the Laves phase requires holding times of 1140 °C and 1160 °C for 59 h and 29 h, respectively. The eutectic (γ + γ′) requires holding times of 1160 °C and 1180 °C for 253 h and 41 h, respectively. The η phase requires holding times of 1180 °C and 1195 °C for 93 h and 14 h, respectively.

### 3.3. Effect of Homogenization on Element Segregation

In order to ensure the stability of the organization and properties of the alloy, in addition to the elimination of the Laves phase, the eutectic (γ + γ′) and the η-phase, it is also necessary to pay attention to the problem of elemental segregation [12,34]. In this study, Ta, Nb and W with serious segregation were analyzed. The residual segregation index *δ* can be used to evaluate the segregation of alloying elements [35]. As shown in Equation (9), *δ* has the following relationship with homogenization temperature *T*, homogenization time *t*, dendrite arm spacing *L* and element diffusion coefficient *D* [9,10,31,32,33]:

(9) $$\delta =\frac{C_{max}^{t}-C_{min}^{t}}{C_{max}^{0}-C_{min}^{0}}=exp\left(-\frac{4\pi^{2}}{L^{2}}Dt\right)$$

where, $C_{max}^{t}$ and $C_{min}^{t}$ are the maximum and minimum values of element concentration after homogenization, respectively. $C_{max}^{0}$ and $C_{min}^{0}$ are the maximum and minimum values of the element concentration of as-cast Ta-GH4151 alloy, respectively. The logarithm of both sides of Equation (9) is obtained [9,10]:

(10) $$\ln\delta =-\frac{4\pi^{2}}{L^{2}}Dt$$

*D* can be obtained from the slope of lnδ vs. *t*. In addition, *D* can also be obtained by the following formula [9,10]:

(11) $$D=D_{0}exp\left(\frac{Q}{RT}\right)$$

where *D*_0_ is the diffusion constant and *Q* is the diffusion activation energy. The logarithm of both sides of Equation (11) is obtained [9,10]:

(12) $$lnD={lnD}_{0}-\frac{Q}{RT}$$

Therefore, *Q* is obtained by the slope of ln*D* vs. 1/*T*.

**Figure 11:**
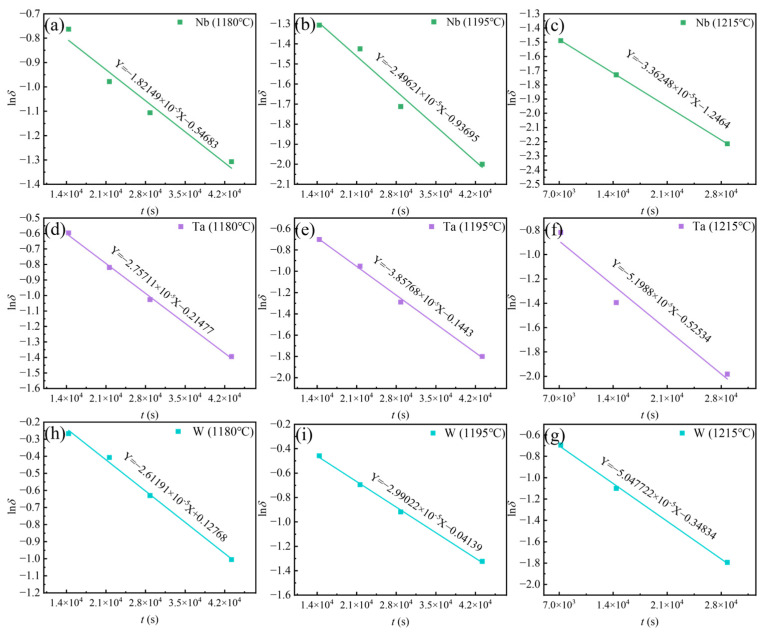
Linear relationship between lnδ vs. t for (**a**) Nb at 1180 °C, (**b**) Nb at 1195 °C, (**c**) Nb at 1215 °C, (**d**) Ta at 1180 °C, (**e**) Ta at 1195 °C, (**f**) Ta at 1215 °C, (**h**) W at 1180 °C, (**i**) W at 1195 °C, (**g**) W at 1215 °C.

Table 4 shows the data of element content and residual segregation coefficient obtained by EPMA. Figure 11 shows the *D* calculated by fitting the linear relationship between ln*δ* and *t*. The residual segregation coefficient equations of W, Ta, and Nb elements at different temperatures are shown in Equations (13)–(21). As the homogenization temperature increases, the diffusion coefficient *D* of the element increases and the required homogenization time is shorter. However, a homogenization temperature that is too high may lead to grain boundary burning, which is an undesired material degradation phenomenon. Therefore, this paper chooses 1195 °C for a long time rather than 1215 °C for a short time to avoid the risk of grain boundary burning. According to the above analysis, the homogenization of as-cast Ta-GH4151 alloy can be divided into three stages:

(1) The temperature of the first stage is 1160 °C: At this temperature, most of the Laves phase and a small amount of the eutectic (γ + γ′) can be eliminated while avoiding the production of caverns.

(2) The temperature of the second stage is 1180 °C: At this stage, the Laves phase and the eutectic (γ + γ′) can be completely eliminated, and the content of the η phase can be significantly reduced.

(3) The temperature of the third stage is 1195 °C. At this stage, the η phase can be completely eliminated, and the uniform diffusion of elements can be promoted through long-term heat preservation to complete the homogenization process.

(13) $$1180\;{}^{\circ}C:\;\delta_{W}=exp\left(-\frac{4\pi^{2}}{0.006134^{2}}2.489\times 10^{-11}t\right)$$

(14) $$1180\;{}^{\circ}C:\;\delta_{Ta}=exp\left(-\frac{4\pi^{2}}{0.006134^{2}}2.628\times 10^{-11}t\right)$$

(15) $$1180\;{}^{\circ}C:\;\delta_{Nb}=exp\left(-\frac{4\pi^{2}}{0.006134^{2}}1.736\times 10^{-11}t\right)$$

(16) $$1195\;{}^{\circ}C:\;\delta_{W}=exp\left(-\frac{4\pi^{2}}{0.006134^{2}}2.85\times 10^{-11}t\right)$$

(17) $$1195\;{}^{\circ}C:\;\delta_{Ta}=exp\left(-\frac{4\pi^{2}}{0.006134^{2}}3.68\times 10^{-11}t\right)$$

(18) $$1195\;{}^{\circ}C:\;\delta_{Nb}=exp\left(-\frac{4\pi^{2}}{0.006134^{2}}2.38\times 10^{-11}t\right)$$

(19) $$1215\;{}^{\circ}C:\;\delta_{W}=exp\left(-\frac{4\pi^{2}}{0.006134^{2}}4.810\times 10^{-11}t\right)$$

(20) $$1215\;{}^{\circ}C:\;\delta_{Ta}=exp\left(-\frac{4\pi^{2}}{0.006134^{2}}4.955\times 10^{-11}t\right)$$

(21) $$1215\;{}^{\circ}C:\;\delta_{Nb}=exp\left(-\frac{4\pi^{2}}{0.006134^{2}}3.205\times 10^{-11}t\right)$$

**Figure 12:**
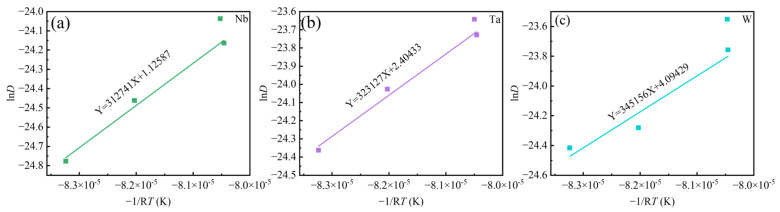
The linear relationship between lnD vs. (−1/RT) for (**a**) Nb, (**b**) Ta, (**c**) W.

Figure 12 illustrates the linear relationship between ln*D* vs. 1/*T*. It is reported that the diffusion activation energy of Ta in Ti is *Q*_Ta/Ti_ = 318 kJ/mol [36], the diffusion activation energy of Ta in Ni is *Q*_Ta/Ni_ =270 kJ/mol [37], the diffusion activation energy of Nb in Ni is *Q*_Nb/Ni_ = 202 kJ/mol [38], the diffusion activation energy of W in Ni is *Q*_W/Ni_ = 261 kJ/mol [37]. Jia et al. [10] reported that the diffusion activation energy of Nb and W in GH4151 alloy is *Q*_Nb_ = 227 kJ/mol, *Q*_W_ = 274 kJ/mol. Li et al. [12] reported that the diffusion activation energy of Nb in GH4151 alloy is *Q*_Nb_ = 252.1 kJ/mol. In this study, the diffusion activation energy of Nb, Ta, and W is *Q*_Nb_ = 313 kJ/mol, *Q*_Ta_ = 323 kJ/mol, and *Q*_W_ = 345 kJ/mol, respectively. These values are higher than those in GH4151 alloy. It suggests that the addition of Ta increases the diffusion activation energy of Nb, Ta, and W, making elemental diffusion more difficult.

**Figure 13:**
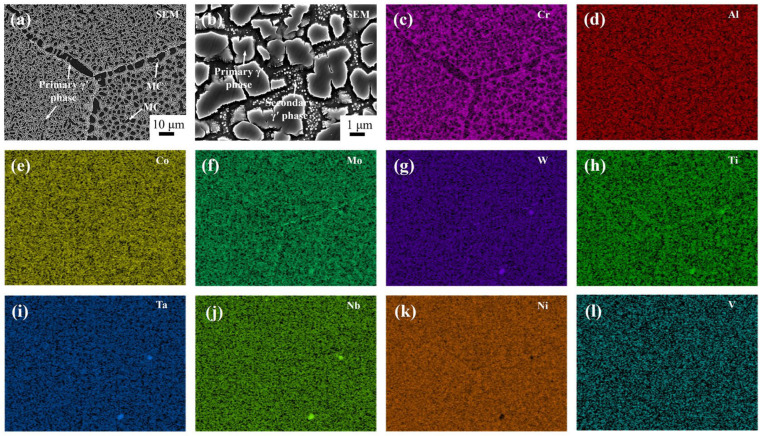
Microscopic structure by SEM (**a**,**b**) and Dendritic structure by EDS (**c**–**l**) of homogenization Ta-GH4151 alloy. (Where (**a**,**b**) are with a magnification of 1000× and 10,000× respectively).

Figure 13 shows the microstructure of Ta-GH4151 alloy after homogenization. Following three-stage homogenization, the microstructure of Ta-GH4151 alloy consists of massive MC phase distributed at grain boundaries and intracrystalline, micron-sized primary irregular γ′ phase distributed at grain boundaries and intracrystalline, and granular secondary γ′ phase distributed in intracrystalline. The segregation secondary phase and element segregation produced during solidification have been effectively eliminated.

### 3.4. Hot Compression Behavior of Homogenized Ta-GH4151 Alloy

**Figure 14:**
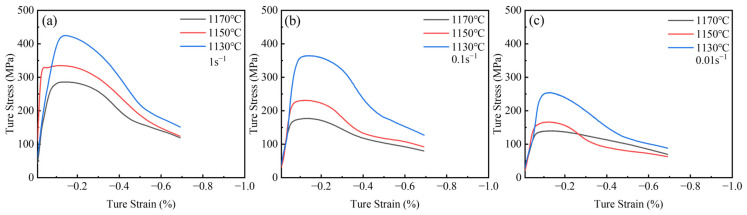
True stress-true strain curves for (**a**) strain rate of 1 s^−1^, (**b**) strain rate of 0.1 s^−1^ and (**c**) strain rate of 0.01 s^−1^ hot compressed Ta-GH4151 alloy under different temperatures.

Homogenization treatment enhances the thermos-plasticity of the alloy. MTS experiments were conducted to evaluate the impact of homogenization on the machinability of Ta-GH4151 alloy. Figure 14 shows the true stress-strain curves of Ta-GH4151 alloy at 1170–1130 °C, 0.01–1 s^−1^ and 50% strain. As strain increases, all samples exhibit work hardening followed by dynamic softening. At a constant temperature, the increase in strain rate leads to the increase of flow stress. It is attributed to the rapid accumulation of deformation energy stored in the grains and massive multiplication and tangle of dislocations at high strain rates. The dynamic softening rate is less than the dislocation multiplication, leading to an increase in flow stress. At a constant strain rate, the increase in deformation temperature leads to a decrease in flow stress. The increase in temperature enhances the diffusion driving force of atoms and vacancies so that more slip systems can be activated during the deformation process. At the same time, the increase in grain boundary migration rate reduces the critical strain of dynamic recrystallization and strengthens the dynamic softening effect, thus reducing the flow stress.

The constitutive equation of high-temperature hot deformation of Ta-GH4151 alloy was developed using the Arrhenius model with the Zener–Holloman coefficient. The correlation and parameter determination methods are shown in Equations (22)–(28) [39,40,41]:

(22) $$\dot{\varepsilon }=\left\{\begin{array}{c} A{\left(sinh\alpha \right)}^{n}exp\left(-\frac{Q\prime }{RT}\right)\\ A^{\prime }\sigma^{n^{\prime }}exp\left(-\frac{Q\prime }{RT}\right)\\ A^{\prime \prime }exp\left(\beta \sigma \right)exp\left(-\frac{Q\prime }{RT}\right) \end{array}\right\}$$

(23) $$Z=\dot{\varepsilon }exp\left(\frac{Q\prime }{RT}\right)=A{\left[sinh\left(\alpha \sigma_{p}\right)\right]}^{n}$$

(24) $$\alpha =\frac{\beta }{n^{\prime }}$$

(25) $$\dot{\varepsilon }=A^{\prime }\sigma^{n^{\prime }}exp\left(-\frac{Q\prime }{RT}\right)$$

(26) $$\dot{\varepsilon }=A^{\prime \prime }exp\left(\beta \sigma \right)exp\left(-\frac{Q\prime }{RT}\right)$$

(27) $$ln\dot{\varepsilon }+\frac{Q\prime }{RT}=lnA+nln\left[sinh\alpha \sigma_{p}\right]$$

(28) Q′=R∙n∙b

where R = 8.314 J/(mol·K) is the gas constant; *Q*′ is the deformation activation energy; *A*, *A*′, *A*″, *β*, *α* and *b* is the material constant; *σ* is the flow stress; $\sigma_{p}$ is the peak stress; *n* and *n*′ is the stress index.

**Figure 15:**
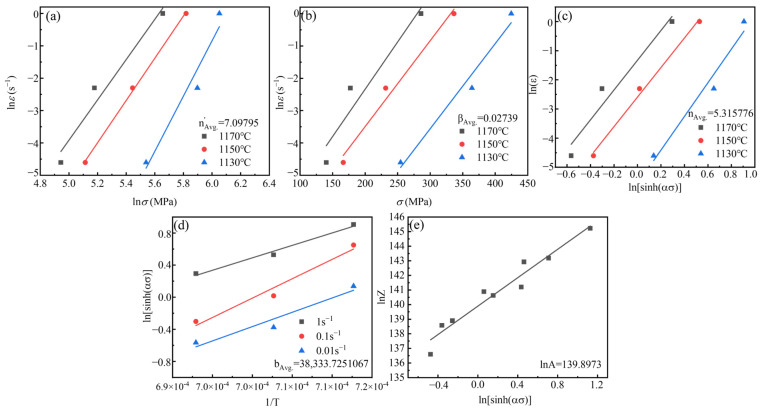
The functional relationship between different parameters, (**a**) $ln\dot{\varepsilon }$ and lnσ, (**b**) $ln\dot{\varepsilon }$ and σ, (**c**) $ln\dot{\varepsilon }$ and $ln\left[sinh\alpha \sigma \right]$, (**d**) $ln\left[sinh\alpha \sigma \right]$ and 1/T, (**e**) lnZ and $ln\left[sinh\alpha \sigma \right]$.

As shown in Figure 15, the hot compression parameters of Ta-GH4151 alloy were obtained by linear fitting of hot compression data: *α* = 0.00386, *n*′ = 7.09795, *β* = 0.02739, *n* = 5.315776, *b* = 388,333.7251067, ln*A =* 139.8973. The thermal activation energy of homogenized Ta-GH4151 alloy is *Q*′_Ta-GH4151_ = 1694.173 kJ/mol. Therefore, the thermal compression constitutive equation of Ta-GH4151 alloy can be expressed as:

(29) $$\dot{\varepsilon }=5.70986\times 10^{60}{\left[sinh\left(0.00386\sigma \right)\right]}^{5.31578}∙exp\left(-\frac{1694173}{RT}\right)$$

To predict the optimized hot working process interval in the hot forming process of Ta-GH4151 alloy, the hot processing map of Ta-GH4151 alloy was constructed based on the dynamic material model theory (DMM). The relationship between the power dissipation *η* and the strain rate sensitivity index *m* is as follows [42,43]:

(30) $$\eta =\frac{J}{J_{max}}=\frac{2m}{m+1}$$

The flow instability criterion proposed by Prasad et al. [44] can be expressed as:

(31) $$\xi \left(\dot{\varepsilon }\right)=\frac{\partial ln\left(\frac{m}{m+1}\right)}{\partial ln\left(\dot{\varepsilon }\right)}+m<0$$

**Figure 16:**
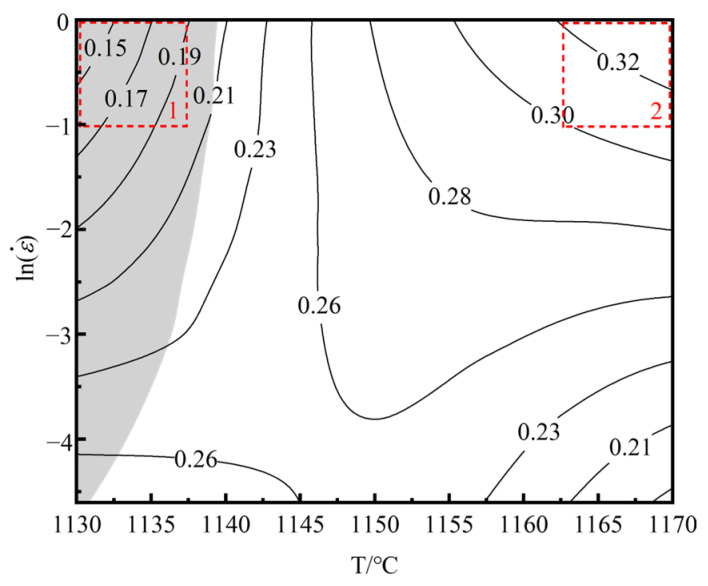
Hot processing map of Ta-GH4151 alloy under the condition of 50% strain.

The hot processing map of the homogenized Ta-GH4151 alloy was obtained by superimposing the power dissipation map and the flow instability map, as shown in Figure 16. The shadow area is identified as the flow instability area, which is characterized by $\xi \left(\dot{\varepsilon }<0\right)$. By analyzing the different microstructures in the thermal processing map, we identified two main regions of thermal deformation mechanisms.

**Figure 17:**
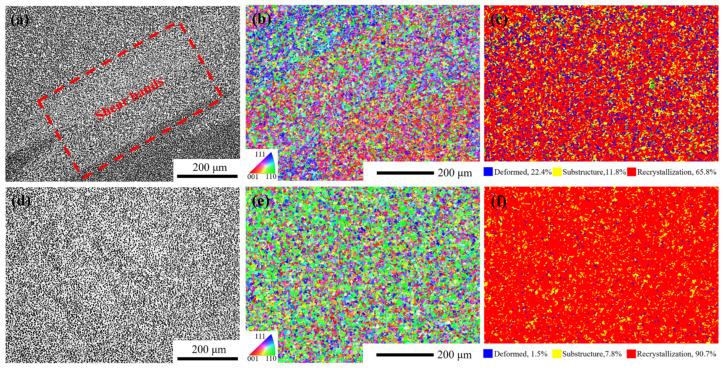
Microstructures under different hot compression parameters. (**a**) optical microstructure at 1130 °C/s^−1^, (**b**) inverse pole figure at 1130 °C/s^−1^, (**c**) recrystallization fraction diagram at 1130 °C/s^−1^, (**d**) optical microstructure at 1170 °C/s^−1^, (**e**) inverse pole figure at 1170 °C/s^−1^, (**f**) recrystallization fraction diagram at 1170 °C/s^−1^.

Region 1: The deformation temperature range is 1130–1140 °C, and the strain rate is 0.01–1 s^−1^. In this region, $\xi \left(\dot{\varepsilon }\right)$ < 0. This region is unstable due to the deformation of alloy plastic. The main instability mechanisms of the alloy during hot deformation include adiabatic shear bands, local plastic flow and cracking [45]. As shown in Figure 17a–c, the recrystallization fraction of the alloy is only 65.8% at 1130 °C/1 s^−1^. Under this condition, the main instability mode of the alloy is the formation of adiabatic shear bands, which usually occur at low temperatures or high strain rates. Due to the short deformation time, the sample is approximately in an adiabatic state, resulting in the failure to effectively release the thermal energy generated during the deformation process, thus contributing to the formation of adiabatic shear bands.

Region 2: The deformation temperature range is 1160–1170 °C, and the strain rate is 0.3^−1^–1 s^−1^. In this region, *η* ≥ 0.3, $\xi \left(\dot{\varepsilon }\right)$ > 0. It is a safe deformation region with high power dissipation efficiency. As shown in Figure 17d,e, taking the hot deformation at 1170 °C/1 s^−1^ as an example, the recrystallization fraction of the alloy in this region exceeds 90%, and the grains do not grow significantly. The hot compression microstructure is mainly composed of fully dynamic recrystallized grains with uniform microstructure and no macroscopic and microscopic defects.

## 4. Discussion

According to the JMAK re-dissolution kinetics Equations (3)–(8), in order to completely eliminate the Laves phase in the as-cast Ta-GH4151 alloy, it is necessary to hold at 1140 °C for 59 h or at 1160 °C for 29 h. In order to completely eliminate the eutectic (γ + γ′), it needs to be kept at 1160 °C for 253 h or at 1180 °C for 41 h. In order to completely eliminate the η phase, it needs to be kept at 1180 °C for 93 h or at 1195 °C for 14 h. In contrast, Li et al. [9] found that the Laves phase can be completely eliminated in the GH4151 alloy without Ta only by holding it at 1140 °C for 10 h. Jia et al. [10] showed that in the GH4151 alloy without Ta, the elimination of eutectic (γ + γ′) needs to be kept at 1180 °C for 28 h, and all precipitated phases can be eliminated at this temperature for 50 h. It can be seen that the addition of Ta significantly increases the difficulty of homogenization of the alloy and increases the temperature of complete dissolution of the Laves phase, eutectic (γ + γ′) and η phase. In fact, Ta is one of the forming elements of these three phases, which can be confirmed by the EDS test results in Table 3. Compared with elements such as Al, Ti and Nb, Ta has a higher atomic number, so its diffusion rate is relatively low. Specifically, in Ta-GH4151 alloy, the diffusion activation energy of Ta is *Q*_Ta_ = 323 kJ/mol (As shown in Figure 12), which is higher than that of Laves phase, eutectic (γ + γ′) and η phase forming elements Ti and Nb without Ta (*Q*_Nb_ = 274.2 kJ/mol, *Q*_Ti_ = 265.2 kJ/mol) [10]. Therefore, we can think that the addition of Ta increases the stability of the three precipitated phases, resulting in increased difficulty in re-dissolution. However, we still addressed the segregation issue of the Ta-GH4151 alloy using three-stage homogenization, and the homogenized microstructure is presented in Figure 13.

In addition, the hot deformation activation energy of Ta-GH4151 alloy under 1130–1170 °C/0.01–1 s^−1^ is *Q*′_Ta-GH4151_ = 1694.173 kJ/mol, with the optimal hot processing temperature and strain rate ranges being 1160–1170 °C and 0.3–1 s⁻^1^, respectively. According to Section 3.4, the study shows that the thermal activation energy *Q*′ and the optimum hot deformation temperature of Ta-GH4151h alloy are significantly higher than those reported in the literature, while the optimum hot deformation range is significantly lower than that reported in the literature. For example, the thermal activation energy *Q*′ of Waspaloy alloy is 630.170 kJ/mol, and the optimum hot deformation range is 1025–1150 °C [19]. The thermal activation energy *Q*′ value of GH4720Li alloy is 1117.9664 kJ/mol [46]; the thermal activation energy *Q*′ value of GH4151 alloy in this study is 1166.996 J/mol, and the optimum hot deformation range is 1100–1135 °C [47]. It can be considered that the addition of Ta significantly increases the optimum hot deformation temperature and activation energy (Q′). This is mainly attributed to the following three factors: First, the addition of Ta increases the re-dissolution temperature of the primary γ′ phase and increases the range of the two-phase region [16], thereby increasing the difficulty of hot deformation. Secondly, the addition of Ta increases the content of the primary γ′ phase [17,18]. Since the strength of the γ′ phase at high temperatures is higher than that of the matrix, the deformation resistance of the alloy is enhanced. Finally, as a precipitation-strengthening element, Ta mainly replaces the position of Al in Ni_3_Al, resulting in a small amount of elements being squeezed into the matrix [6,7], thereby increasing the solid solubility of the matrix and further enhancing the strength of the alloy. Although Ta increases the difficulty of thermal processing, there is still a theoretical thermal deformation safety interval for Ta-GH4151 alloy. At present, our research group has successfully realized the hot extrusion of Ta-GH4151 alloy and will release relevant research results in the future.

## 5. Conclusions

In this paper, the as-cast microstructure, microsegregation, the kinetics of the secondary precipitation phase and thermal deformation behavior in Ta-GH4151 alloy were studied, and the main conclusions are as follows.

1. The as-cast Ta-GH4151 alloy exhibits a complex microstructure with significant element segregation. Elements such as Ti, Ta, Nb, and Mo predominantly reside in the interdendritic regions, displaying negative segregation, while Cr and W are enriched in the dendritic arm regions, showing positive segregation. This segregation pattern is attributed to the non-equilibrium solidification process, where the addition of Ta exacerbates the segregation of Nb, Ta, and W, thereby influencing the alloy’s microstructure and properties.

2. The study establishes the initial dissolution temperatures for the Laves phase (1140–1150 °C), eutectic (γ + γ′) (1150–1160 °C), and η phase (1170–1180 °C). The diffusion activation energies for Nb, Ta, and W are determined to be 313 kJ/mol, 323 kJ/mol, and 345 kJ/mol, respectively. These findings indicate that the addition of Ta increases the diffusion activation energy of these elements, making elemental diffusion more challenging and necessitating a three-stage homogenization process at 1160 °C, 1180 °C, and 1195 °C to achieve effective homogenization.

3. The homogenized Ta-GH4151 alloy has a hot compression activation energy of 1694.173 kJ/mol, indicating a high resistance to hot deformation. The optimal hot processing range is identified as 1160–1170 °C with a strain rate of 0.3 s⁻¹–1 s⁻¹. Despite the increased difficulty in hot processing due to Ta addition, the alloy still possesses a theoretical hot deformation safety interval, and successful hot extrusion has been achieved, demonstrating the feasibility of industrial application.

## Figures and Tables

**Table 1:** Nominal composition of Ta-GH4151 alloy (wt.%).

Alloy	C	Co	Cr	Mo	W	Al	Ti	Nb	Ta	Ni
Ta-GH4151	0.04	15.0	11.0	4.5	3.0	3.0	2.5	33.0	3.0	Bal.

**Table 2:** Hot compression parameters of homogenized Ta-GH4151 alloy.

Temperature/°C	Heating Rate/(°C/s)	Holding Time/s	Deformation Rate/s^−1^	Strain/%
1130, 1150, 1170	5	600	0.01, 0.1, 1	50

**Table 3:** Composition of secondary precipitates phase in as-cast Ta-GH4151 alloy by EPMA (wt.%).

	W	Mo	Co	Cr	Al	Ti	Nb	Ta	Ni	C
γ + γ′	1.6 ± 0.6	0.5 ± 0.5	11.5 ± 0.6	3.6 ± 0.3	4.8 ± 0.2	6.0 ± 0.3	3.5 ± 0.5	5.5 ± 0.6	59.3 ± 1.1	3.6 ± 0.7
η	2.3 ± 0.7	1.5 ± 0.6	12.4 ± 0.7	3.3 ± 0.3	2.9 ± 0.2	4.7 ± 0.3	7.5 ± 0.6	6.5 ± 0.6	54.0 ± 1.2	2.7 ± 0.8
Laves	5.2 ± 0.6	15.5 ± 0.9	16.6 ± 0.7	16.1 ± 0.6	0.6 ± 0.1	1.4 ± 0.2	16.0 ± 0.8	5.8 ± 0.6	19.5 ± 0.8	3.3 ± 0.9
MC	4.0 ± 0.8	1.0 ± 0.5	1.3 ± 0.4	0.5 ± 0.3	0	13.2 ± 0.5	34.9 ± 1.0	30.3 ± 0.9	3.0 ± 0.6	12.0 ± 1.1

**Table 4:** Element content and residual segregation index (*δ*) during homogenization (wt.%).

	Nb			W			Ta		
	$C_{min}^{t}$	$C_{max}^{t}$	*δ*	$C_{min}^{t}$	$C_{max}^{t}$	*δ*	$C_{min}^{t}$	$C_{max}^{t}$	*δ*
As cast	1.4	8.0	1.000	1.7	4.7	1.000	1.6	5.2	1.000
1180 °C/4 h	1.6	4.7	0.466	2.2	4.5	0.756	1.6	3.6	0.551
1180 °C/6 h	2.1	4.6	0.376	2.4	4.4	0.665	2.0	3.6	0.441
1180 °C/8 h	2.2	4.4	0.331	2.7	4.3	0.532	2.2	3.5	0.358
1180 °C/12 h	2.2	4.0	0.271	2.8	3.8	0.366	2.4	3.3	0.250
1195 °C/4 h	1.9	3.7	0.271	2.6	4.5	0.632	1.7	3.5	0.496
1195 °C/6 h	2.1	3.7	0.241	2.6	4.1	0.499	2.0	3.4	0.386
1195 °C/8 h	2.2	3.4	0.180	2.8	4.0	0.399	2.2	3.2	0.275
1195 °C/12 h	2.2	3.1	0.135	3.0	3.8	0.266	2.4	3.0	0.165
1215 °C/2 h	2.1	3.6	0.226	2.7	4.2	0.499	2.0	3.6	0.441
1215 °C/4 h	2.1	3.4	0.177	2.9	3.9	0.333	2.4	3.3	0.248
1215 °C/8 h	2.3	3.1	0.109	3.1	3.6	0.166	2.6	3.1	0.138

## Data Availability

The original contributions presented in this study are included in the article. Further inquiries can be directed to the corresponding author.
